# Case report: *In vivo* detection of neutrophil extracellular traps in a dog with thrombosis induced by bacterial vasculitis

**DOI:** 10.3389/fvets.2025.1470605

**Published:** 2025-02-12

**Authors:** Ju-Yun Kim, Hyun-Jung Han

**Affiliations:** Department of Veterinary Emergency and Critical Care, College of Veterinary Medicine, Konkuk University, Seoul, Republic of Korea

**Keywords:** NETosis, thrombosis, bacterial vasculitis, dog, case report

## Abstract

This case report describes NETosis as a cause of thrombosis in an 18.3 kg, 8-year-old intact male mixed-breed dog with bacterial vasculitis. The dog presented with sudden paresis of the thoracic limb, characterized by cyanosis, absent arterial pulse, and decreased peripheral blood glucose levels. Doppler ultrasound confirmed thrombosis in the dorsal common digital artery. Histopathology post-amputation revealed bacterial vasculitis, thrombosis, and infarction, with immunohistochemical staining identifying extracellular citrullinated histone H3 (CitH3), indicative of NETs involvement. Treatment included antibiotics, pentoxifylline, and anticoagulants, showing transient improvement before disease progression and euthanasia due to respiratory signs. These findings suggest NETs as a potential therapeutic target for bacterial vasculitis in similar cases.

## 1 Introduction

Neutrophil extracellular traps (NETs) are an innate immune response to infections, including mechanisms such as phagocytosis and degranulation, and serve as one of the defense mechanisms of neutrophils (1, 2). NETs are released through a programmed cell death mechanism known as NETosis, first described by Brinkmann et al. in 2004 as a means for neutrophils to trap and kill bacteria (3, 4). Neutrophils selectively release NETs under specific conditions, such as detecting pathogen size and responding to larger pathogens where phagocytosis is limited (5). NETs consist of chromatin, granules, and some cytoplasmic components, exhibiting excellent antibacterial activity and contributing to immune defense (6, 7). In particular, granular components like myeloperoxidase (MPO), alpha-defensins, elastase (NE), cathepsin G, and lactoferrin possess bactericidal properties capable of eliminating microorganisms and their virulence factors (8, 9).

Bacterial vasculitis is characterized by inflammatory responses that damage blood vessel walls and increase susceptibility to thrombosis (10). NETs are increasingly recognized as key contributors to immunothrombosis, a term describing the interplay between NETs and coagulation, which suggests that NETs can promote thrombus formation (11, 12). Histone components within NETs have been shown to promote platelet aggregation, further supporting the idea that an abundance of NETs contributes to thrombotic events (13, 14). In humans, excessive NET formation has been linked to small-vessel vasculitis and thromboembolic conditions, where NETs cause significant endothelial damage and amplify inflammation (15). There is evidence that NETs may exacerbate vascular injury by promoting inflammation and thrombosis in vasculitis-related conditions (16).

In veterinary medicine, growing evidence indicates that NETs are involved in thrombus formation. Specifically, NETs appear to play a critical role in thrombosis associated with immune-mediated diseases in cats (17). These findings highlight the potential of targeting NETs as a therapeutic strategy for managing thrombosis induced by bacterial vasculitis.

This case report presents NETosis as a possible cause of systemic thrombosis in a dog diagnosed with necrotizing vasculitis due to bacterial infection. To our knowledge, this is the first case of *in vivo* detection of NETs in a dog with thrombosis caused by bacterial vasculitis, underscoring the potential of NETs as a treatment target for thrombosis induced by bacterial vasculitis in this clinical context.

## 2 Case report

An 8–year–old intact male mixed-breed dog weighing 18.3 kg presented to Konkuk Veterinary Medical Teaching Hospital with acute bilateral thoracic limb paresis. There was no history of trauma, drug exposure, ectoparasite exposure, travel, or dietary changes. The patient was up to date with routine vaccinations and parasitic prevention in the last 36 months.

Three months prior to the onset of thoracic limb paresis, the dog was treated by a primary care veterinarian for chronic diarrhea, hematochezia, anorexia, and weight loss. The dog was diagnosed with inflammatory bowel disease (IBD) based on intestinal wall thickening, mesenteric lymphadenopathy on abdominal ultrasound, and negative responses to dietary and antibiotic trials. IBD was managed with a hypoallergenic diet, metronidazole (12.5 mg/kg, PO, q12h) for 7 days, prednisolone (1 mg/kg, PO, q12h), and cyclosporine (2.5 mg/kg, PO, q12h). The patient showed a positive response to treatment, with prednisolone and cyclosporine tapered over 1 month, while the hypoallergenic diet was maintained. However, the patient was hospitalized 10 days prior to presentation due to the recurrence of lethargy and hematochezia. The treatment was resumed at the local animal hospital, including metronidazole (12.5 mg/kg, intravenous [IV], q12h), prednisolone (0.5 mg/kg, PO, q12h), and cyclosporine (2.5 mg/kg, PO, q12h). On the second day of hospitalization at that facility, acute bilateral thoracic limb paresis was identified, and it progressed gradually, leading to the transfer to a tertiary hospital on day 10 of hospitalization.

**Figure 1:**
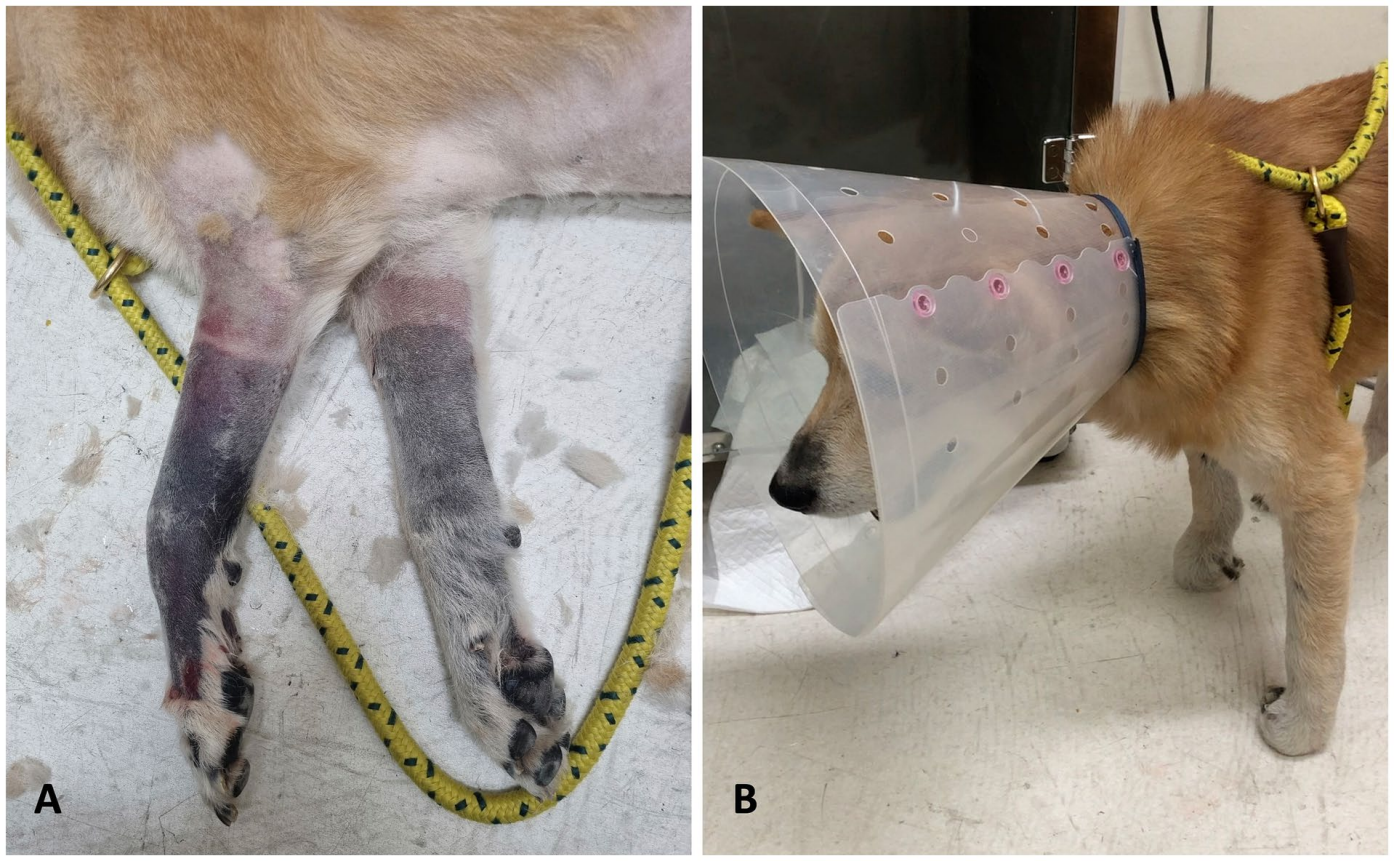
Thoracic limbs appearance at the time of admission. **(A)** After clipping, cyanotic changes with well–defined boundaries were observed on both thoracic limbs. **(B)** Physical examination revealed ambulatory thoracic limb paresis and proprioceptive ataxia.

On presentation, the dog was alert and responsive, with normal vital parameters including temperature, heart rate, and respiratory rate, as well as a normal systolic blood pressure. Physical examination revealed severe discoloration of both thoracic limbs from the mid-radius to the digits, with coolness upon palpation in this area. An absence of arterial pulse beyond the affected region and severe pain extending to the mid-radius were noted (Figure 1A). Neurological assessment revealed severe thoracic limb paresis, proprioceptive ataxia, and postural deficits (Figure 1B), along with decreased spinal reflexes in the thoracic limbs, while normal postural reactions and spinal reflexes were observed in the pelvic limbs. The presence of ischemic cutaneous lesions suggested localized vascular compromise, while the absence of pronounced muscle atrophy was inconsistent with a chronic peripheral nerve abnormality. These findings, combined with the clinical presentation, pointed to acute peripheral nerve involvement, specifically affecting the musculocutaneous and radial nerves branching from the C6–T2 spinal segment.

The in-house complete blood count revealed moderate anemia (26.1%, reference range: 36.0–60.0%), severe leukocytosis (51.7 K/mcL, reference range: 6.0–17.0 K/mcL) characterized by mature neutrophilia (43.5 K/mcL, reference range: 3.0–11.5 K/mcL) with a left shift (2.6 K/mcL, reference range: 0.0–0.5 K/mcL), monocytosis (1.7 K/mcL, reference range: 0.1–1.5 K/mcL), and lymphocytosis (6.3 K/mcL, reference range: 1.0–5.0 K/mcL).^1^ White blood cell morphology revealed moderate toxic changes in many neutrophils. The in-house serum biochemistry tests, conducted immediately after collecting the blood sample from the jugular vein, revealed a normal glucose concentration (96 mg/dL, reference range: 70–143 mg/dL) and elevated concentrations of lactate (5.6 mmol/L, reference range: < 2.0 mmol/L), alanine transaminase (ALT; 165 U/L, reference range: 19–70 U/L), alkaline phosphatase (ALKP; 1,053 U/L, reference range: 0–150 U/L), gamma–glutamyltransferase (GGT; 33 U/L, reference range: 0–13 U/L), and C-reactive protein (CRP; 2.6 mg/dL, reference range: 0.1–1.0 mg/dL).^2^ Additionally, mild hypocalcemia was noted in total calcium (7.8 mg/dL, reference range: 8.5–11.0 mg/dL), while creatine kinase (CK; 113 U/L, reference range: 10–200 U/L) and aspartate aminotransferase (AST; 45 U/L, reference range: 0–50 U/L) were within normal limits. Furthermore, a notable reduction in blood glucose concentration was observed in the thoracic limb samples collected from the paw pads (< 20 mg/dL, reference range: 70–143 mg/dL) compared to the hind limb sample (77 mg/dL, reference range: 70–143 mg/dL) using a point–of–care device^3^. Additionally, plasma lactate concentrations in the thoracic limb samples were notably higher (11.8 mmol/L, reference range, < 2.0 mmol/L) compared to the hind limb samples (6.1 mmol/L) using a chemistry analyzer^b^.

The results of concurrent urinalysis were unremarkable. Coagulation profiles showed elevated D–dimers (4,537 ng/mL; reference range, 50–250 ng/mL)^4^ and hypercoagulability on kaolin–activated thromboelastography (TEG) with an R-value of 1.8 min (reference range, 1.8–8.6 min), a decreased K value of 0.8 min (reference range, 1.3–5.7 min), an increased alpha angle of 78.0 degrees (reference range, 36.9–74.6 degrees), and an increased maximum amplitude (MA) of 75.3 mm (reference range, 42.9–67.9 mm)^5^.

**Figure 2:**
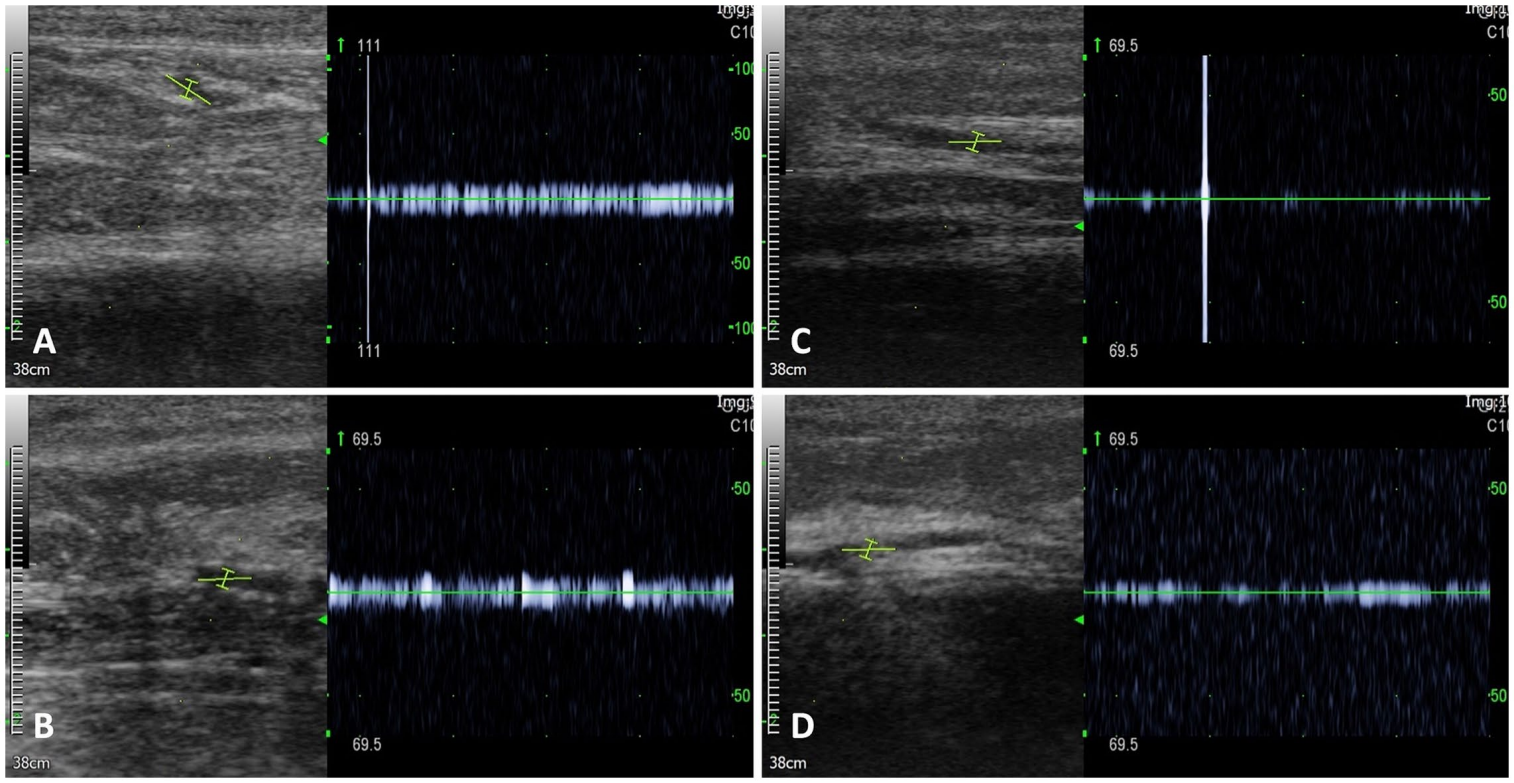
Doppler signals on the thoracic limbs. Doppler signal was identified in the common digital artery of the right thoracic limb **(A)** and left thoracic limb **(B)**. However, the Doppler signal decreased as it progressed to the digital artery of the right thoracic limb **(C)** and left thoracic limb **(D)**.

In-house vector-borne disease/pathogen testing was negative for *Dirofilaria* antigen, *Ehrlichia canis*, *E. ewingii*, *Borrelia burgdorferi*, *Anaplasma phagocytophilum*, and *A. platys.*^6^ Additionally, the IDEXX Canine Tick/Vector Panel and Diarrhea Panel tests for infectious organisms associated with vasculitis were negative. Serum antinuclear antibodies (ANA)^7^ and antineutrophil cytoplasmic antibodies (ANCA)^8^ for autoantibody-associated vasculitis were also negative. Blood cultures from three jugular vein samples collected at different time points showed no bacteremia. Although long-term antibiotic treatment may affect accuracy, the samples were collected just before the next dose of intravenous antibiotics, 12 h after the last administration. Echocardiography to evaluate infective endocarditis presented unremarkable findings. The Doppler signal gradually decreased from the common digital artery to the digital artery and disappeared at the distal part of the digital artery of the thoracic limbs (Figure 2). Radiography of the thoracic, abdominal, forelimb, and hindlimb regions revealed no abnormalities.

On hospitalization day 1, prior to surgery, the patient continued receiving the same medications that had been administered during the recent hospitalization at a local animal hospital. This included metronidazole (12.5 mg/kg, intravenous [IV], q12h)^9^, prednisolone (0.5 mg/kg, PO, q12h)^10^, cyclosporine (2.5 mg/kg, PO, q12h)^11^, along with pantoprazole (1 mg/kg, IV, q12h)^12^ and pentoxifylline (15 mg/kg, PO, q8h)^13^.

The significant progression of tissue necrosis with well-defined boundaries resulted in severe pain with limited recovery potential, prompting us to perform a partial bilateral amputation (mid-radius amputation) on the second day of hospitalization. The patient was premedicated with cefazoline (30 mg/kg, IV)^14^, famotidine (1 mg/kg, IV)^15^, butorphanol (0.2 mg/kg, IV)^16^, and midazolam (0.3 mg/kg, IV)^17^. General anesthesia was induced with propofol (4–6 mg/kg, IV)^18^ and maintained with isoflurane after endotracheal intubation.

**Figure 3:**
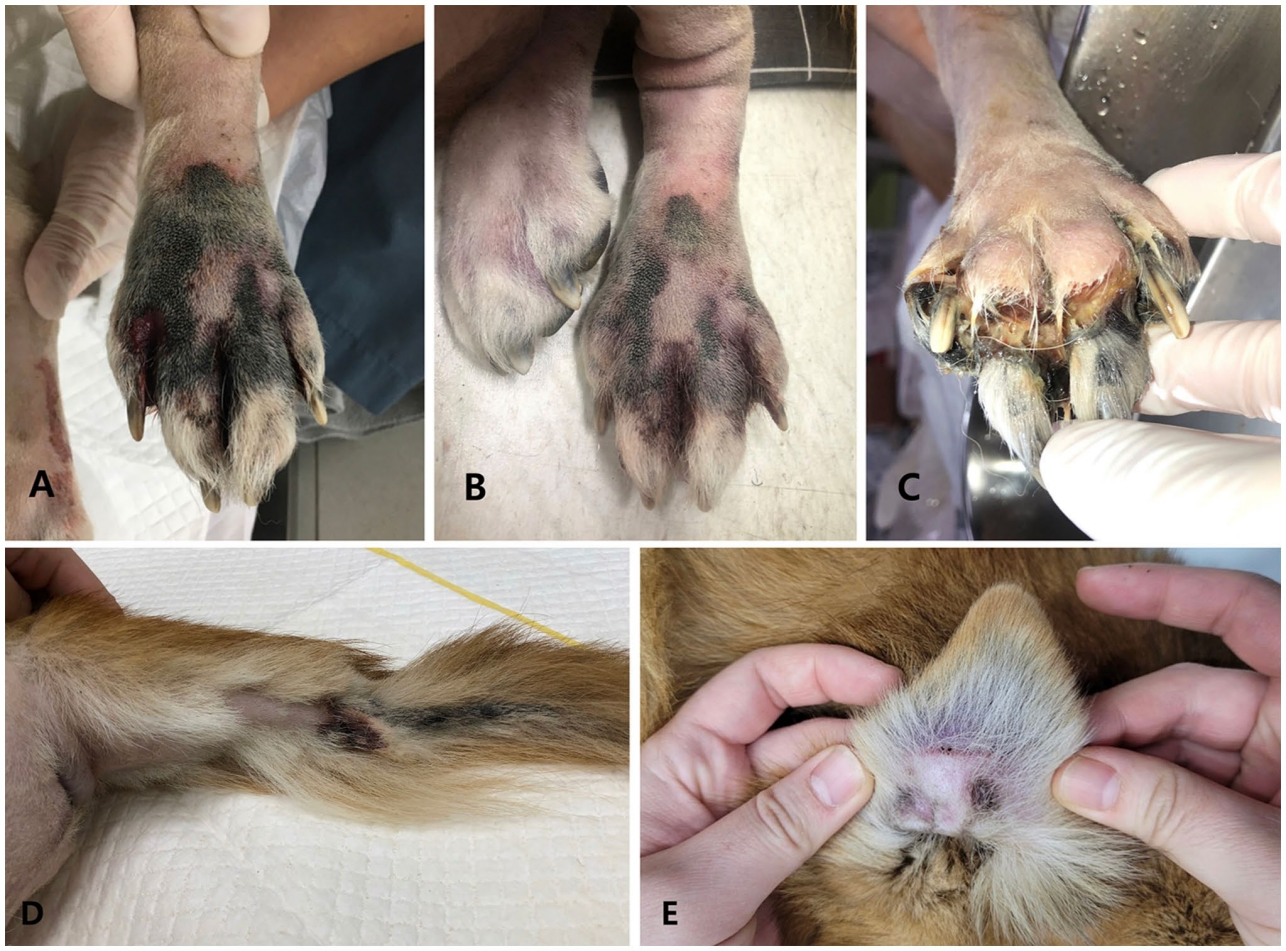
Gross photographs on the left hindlimb **(A,B)**, right hindlimb **(C)**, tail **(D)**, and pinna **(E)**. **(A)** Immediately after surgery, cyanotic color changes, accompanied by coolness and weak arterial pulse, were noted from the distal toes to the metatarsus of the left hindlimb, similar to findings in the thoracic limbs. **(B)** Following empirical treatment for vasculitis, transient improvement was observed in the left hindlimb on day 7 post-surgery as the discoloration of necrotic tissue lightened. Nonetheless, on day 10 post-surgery, the lesion appeared on the distal toes of the right hindlimb **(C)**, and on day 13 post-surgery, it also appeared on the pinna margin **(D)** and the tip of the tail **(E)**.

The dog was positioned in dorsal recumbency for a craniomedial surgical approach to the distal third of the radius and ulna bilaterally. A circumferential transverse incision was made on the skin and musculotendinous tissue of the right thoracic limb, providing a 1 cm margin for the necrotic tissue. The right dorsal common digital artery revealed an intravascular thrombus (Figure 3A). Thrombus-containing vessels were excised and fixed in 10% formalin for histological examination (Figure 3B). Vessel ligation was performed, followed by proximal soft tissue retraction using Senn handheld retractors. Transverse osteotomy of the distal radius and ulna was conducted with an oscillating saw equipped with a 9.2 mm blade, along with saline lavage. The area was irrigated, and muscles were closed with a simple continuous pattern. Subcutaneous tissues and skin were closed routinely. The left thoracic limb underwent osteotomy to the same level, but intravascular thrombosis was not evident. The dog recovered from anesthesia without complications. Postoperative analgesia was provided via bolus administration of fentanyl (0.004 mg/kg, IV)^19^ and lidocaine (0.5 mg/kg, IV)^20^, followed by continuous infusion of fentanyl (0.004 mg/kg/h, IV; see footnote 19) and lidocaine (1.2 mg/kg/h, IV; see footnote 20) for 24 h.

Immediately after surgery, further tissue cyanosis was noted in the left pelvic limb, extending from the distal toes to the middle of the metatarsus. This was accompanied by coolness and a weakened arterial pulse beyond this area toward the distal part (Figure 3A). Given the heightened risk of postoperative infection resulting from immunosuppression, cyclosporine was discontinued postoperatively, and prednisolone was gradually tapered and ultimately ceased over the course of 1 week.

To address potential infections, the patient received empiric broad-spectrum antibiotics, including cefotaxime (80 mg/kg, IV, q8h)^21^ and an adjusted dose of metronidazole (15 mg/kg, IV, q12h; see footnote 9), which had been continued from previous therapy. This decision was made in the absence of culture results to mitigate the risk of infection while awaiting histopathological evaluation. Additionally, the patient was treated with pentoxifylline (15 mg/kg, PO, q8h; see footnote 13), niacinamide (500 mg/dog, PO, q8h)^22^, fish-derived omega-3 fatty acids (50 mg/kg, PO, q24h)^23^, vitamin E (400 units/dog, PO, q24h)^24^, and doxycycline (5 mg/kg, PO, q12h)^25^ for potential unconfirmed rickettsial disease and concurrently for its additive anti-inflammatory effect. Maximum-dose anticoagulant (rivaroxaban, 2 mg/kg, PO, q12h)^26^ and antiplatelet therapy (clopidogrel, 4 mg/kg, PO, q24h)^27^ were initiated 24 h postoperatively to minimize the risk of thromboembolism while reducing the risk of bleeding at the surgical site. Additionally, thermal warming of the affected limbs was implemented. Transient improvement was observed in the left hind limb on postoperative day 7 (Figure 3B), and the patient was discharged for home management on the same day. However, by postoperative day 10, the lesion had progressed to the distal toes of the right hind limb (Figure 3C). Similar to the forelimbs, severe pelvic limb paresis and proprioceptive ataxia were noted in the hind limbs. Neurological assessment revealed postural deficits and diminished spinal reflexes, indicating involvement of the lumbosacral spinal cord (L4–S1). The pronounced paresis, combined with ischemic lesions, suggested localized vascular compromise with potential involvement of the femoral and sciatic nerves. The absence of muscle atrophy further supported the likelihood of an acute process. By postoperative day 13, additional ischemic lesions were observed at the tip of the tail (Figure 3D) and along the pinna margin (Figure 3E). Despite initial improvement, the patient deteriorated and began displaying respiratory signs on postoperative day 34. The owner reported significant difficulties in nursing care, including challenges in administering medications accurately and consistently, managing the patient’s pain and discomfort effectively, and ensuring proper nutrition and hydration. Additionally, the owner’s limited experience in caring for a post-operative patient compounded these challenges. Ultimately, the patient was euthanized at the owner’s request owing to poor prognosis, these nursing difficulties, and financial constraints. Consent was not given for postmortem examination.

**Figure 4:**
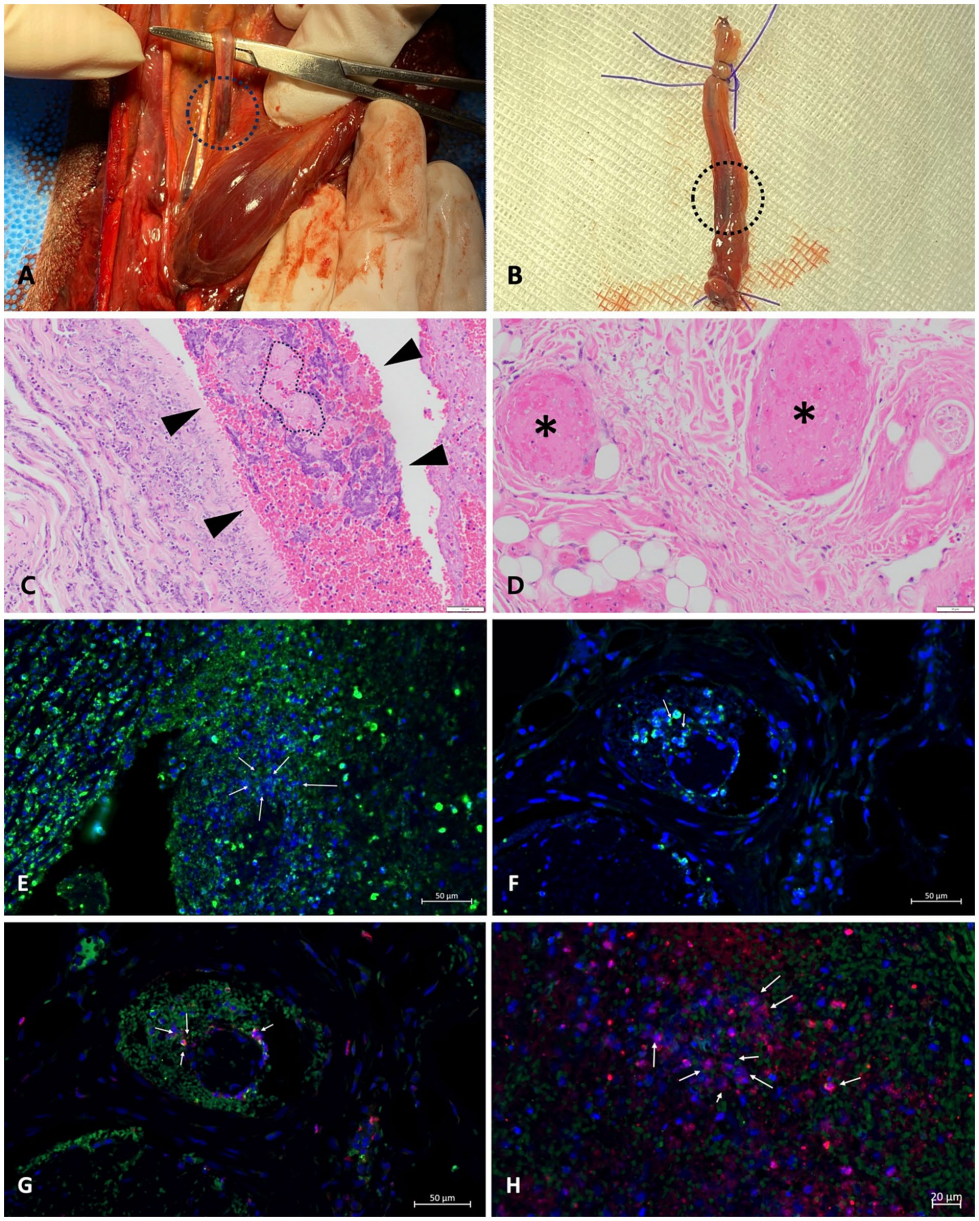
NETs in a thrombus of a dog with septic vasculitis: **(A,B)** macroscopic images taken during surgery; **(C,D)** H&E staining of the tissue of thoracic limbs; **(E–H)** immunofluorescence analysis of thrombus of the right thoracic limb conducted to identify NETs. **(A)** A thrombus (black circle) in the right dorsal common digital artery of the right thoracic limb was confirmed. **(B)** The vessel with thrombi (black circle) of the right thoracic limb was completely excised for histopathological examination. **(C)** The lumen of the artery segment of the right thoracic limb was partially filled with fibrin intermixed with individual to clusters of bacteria. The region highlighted with dashed lines in the vascular area contains fibrin associated with cocci. The light pink color represents the fibrin, while the very small circles indicate the presence of cocci. Numerous purple circles are scattered throughout the fibrin, further illustrating the distribution of cocci in this region. **(D)** Several blood vessels within the subcutis of the left thoracic limb are occluded by fibrin thrombi (* indicates blood vessels affected by fibrin thrombi). **(G,H)** Granular antigens, including another elastase (in green), citrullinated histones (in red), and DNA (in blue), co-localize, presenting NETosis (arrows).

Histopathological evaluation of the affected limb muscles and vessels with thrombi, obtained postoperatively, revealed multifocal segmental neutrophilic and necrotizing vasculitis with thrombosis, intraluminal bacteria, and extensive cutaneous infarction. These findings became available on postoperative day 17 and confirmed the diagnosis. However, no additional changes to the treatment plan were made at that time, as the patient’s ongoing therapies, including maximum-dose anticoagulant and antiplatelet treatments, were deemed appropriate. The primary lesion in the right thoracic limb showed bacterial vasculitis with secondary thrombosis and infarction. Bacteria were scattered within the lumen and arterial walls, consistent with septic vasculitis. Neutrophils infiltrated surrounding nerves and fibroadipose tissue. There was occlusion of blood vessels of the deep dermis in both thoracic limbs with a mixture of fibrin thrombi and clusters of cocci. Morphological characteristics observed with Hematoxylin and Eosin (H&E) staining suggested *Streptococcus* or *Staphylococcus* spp. Tissue cultures were not performed on the limbs either before or after amputation. Furthermore, immunofluorescence analysis of paraffin-embedded tissue samples from thrombi in the collapsed arterial segment of the right thoracic limb confirmed the presence of NETs, identified by the colocalization of myeloperoxidase (MPO) and citrullinated histones (H3Cit) (Figure 4). A detailed protocol for tissue preparation and immunofluorescence analysis, including DAPI counterstaining (concentration and inclusion in the mounting medium) and secondary antibody concentrations, is provided in Supplementary material 1 to facilitate replication. Briefly, the process involved antigen retrieval, tissue permeabilization, and blocking to minimize nonspecific binding. Primary antibodies specific to MPO and H3Cit were applied, followed by species-specific secondary antibodies. Confocal microscopy^28^ was conducted using laser wavelengths optimized for DAPI, MPO, and H3Cit detection (18).

## 3 Discussion

This case describes a dog presenting with limb ischemic necrosis caused by bacterial vasculitis and thrombosis, providing the first veterinary evidence of neutrophil extracellular trap (NET) involvement in an infectious thrombus. Vasculitis is a reactive disease characterized by abnormal immune responses in blood vessels. It is defined by the presence of leukocytes in the vessel wall, resulting in reactive damage to the mural structures, ultimately leading to tissue ischemia and necrosis (19–21). Although its pathophysiology is not fully understood, type III hypersensitivity reactions involving immune complex deposition may play the most significant role, while type I and II hypersensitivity reactions may have potential contributions (20).

In both human and veterinary medicine, vasculitis is primarily categorized as autoimmune (primary vasculitis) or secondary to other underlying conditions (secondary vasculitis) (20–23). Primary vasculitis typically indicates an autoimmune disorder without identifiable infectious agents, while secondary vasculitis can result from factors such as infections, drug reactions, blood transfusions, and flea bites (20–32). Bacterial vasculitis in dogs often links to bacteremia, local injury, bacterial inoculation, intra-abdominal pathologies, and septic emboli, as seen in infective endocarditis (20). In this patient diagnosed with septic vasculitis via histopathological examination, potential causes included gastrointestinal bacterial translocation due to underlying IBD, chronic immunosuppression from previous immunosuppressants, and the use of intravenous catheters. However, there were no clear clinical signs of sepsis, and the blood culture was negative, which may be attributed to antibiotic treatment for enteritis causing false-negative results.

Thromboembolic events and vessel occlusion by fibrin thrombi are common complications of systemic vasculitis associated with sepsis. Inflammatory cytokines such as tumor necrosis factor–*α* and interleukin–1 promote these thrombotic processes by upregulating endothelial tissue factor expression (24, 33, 34). Although severe extremity ischemia due to infectious vasculitis has not been reported in veterinary medicine, it has been documented in human cases. For example, a case report described symmetrical peripheral gangrene caused by vasculitis triggered by Rocky Mountain Spotted Fever. Biopsy results revealed vasculitis with small vessel occlusion and the presence of *Rickettsia rickettsii* on direct immunofluorescence staining and blood cultures. These conditions contributed to a hypercoagulable state, disseminated intravascular coagulation, and hypotension, ultimately leading to distal extremity necrosis and gangrene (35). Systemic vasculitis can disrupt blood flow, resulting in dermatological manifestations such as palpable purpura, plaques, hemorrhagic bullae, wheals, and serpentine papules, often accompanied by pitting edema. While these dermatologic lesions are more commonly observed, thromboembolic events, vascular occlusion, and necrosis can result in atypical manifestations. Concurrent systemic symptoms, including anorexia, depression, malaise, and fever, may also occur (32). As the role of NETs in infectious vasculitis has not been previously reported in veterinary medicine, this canine case report sought to confirm the presence of NETs in infectious vasculitis and thrombosis.

NETs play crucial roles in microbial defense (1). Granular and nuclear antigens exhibit spatial distinction, with granular proteins predominantly localized in the cytoplasm and nuclear proteins associated with chromatin and histones (3). Upon recognition of pathogen-associated molecular patterns (PAMPs) by neutrophil receptors, certain granular proteins like MPO and NE translocate to the nucleus, inducing chromatin decondensation through peptidylarginine deiminase 4 (PAD4)-mediated histone citrullination or direct action of MPO and NE (36–38). These components amalgamate in the cytoplasm to form NETs, which are then released by neutrophils into the extracellular space to ensnare bacteria and restrict their dissemination (1, 39). In addition to enhancing microbial defense, NETs are formed through the interaction of LPS-activated platelets and neutrophils, which not only facilitate local bacterial trapping and improve bacterial clearance in wound infections but may also contribute to thrombosis (11). However, excessive NET formation may contribute to thrombosis. While the signaling pathways inducing NETs in deep vein thrombosis are unclear, extracellular DNA traps act as scaffolds for platelet binding and aggregation, facilitating thrombus formation (12). Histones, especially citrullinated forms crucial for NET induction, promote platelet aggregation and microvascular thrombosis in animal models (13).

Multiple techniques are available to detect and quantify NETs in tissue, including immunofluorescence microscopy and specific NET markers, such as free DNA, IL-17, and PR-39 (40–44). In this study, immunofluorescence microscopy was performed on paraffin-embedded tissue sections of thrombi to assess NET markers. Specific antibodies against granular antigens (e.g., MPO, elastase) and histone-DNA complexes confirmed the presence of NETs (18). Additionally, histone citrullination, a key process in chromatin decondensation, was detected, further supporting NET formation (38). This patient exhibited peripheral ischemic necrosis in the extremities, pinna, and tail, which was diagnosed as thrombosis secondary to bacterial vasculitis. Histological analysis revealed colocalization of immunofluorescent signals for nuclear and granular NET components within the thrombi. Extensive histone citrullination in the thrombus indicated an immune response to bacterial vasculitis, triggering excessive NET formation and contributing to microvascular thrombus development. This aligns with previous studies demonstrating the role of NETs, particularly histones, in promoting thrombosis (1, 45).

The capacity to induce NETosis varies among bacterial species, with *Staphylococcus aureus* being a particularly potent inducer (46). *S. aureus,* a Gram-positive bacterium, is known to cause various infections and is frequently isolated from chronic wound infections, such as osteomyelitis and ulcers, in humans and animals (47, 48). In humans, *S. aureus*-associated vasculitis often results from nasal carriage or hematogenous spread, including bacterial endocarditis (49). To evade immune responses, *S. aureus* employs strategies such as nuclease production to escape NETs and inhibit neutrophil recognition (50–55). Additionally, *Staphylococcal* Protein A plays a critical role in evading phagocytosis by preventing opsonization and inducing B-cell apoptosis (54, 56–58). Recent studies have demonstrated a positive correlation between Protein A secretion and NETosis induction by *S. aureus* (46). Furthermore, NETs have been observed in cerebrospinal fluid during *Streptococcus suis* infections in piglets, where the virulent strain causing meningitis induced NETs within 2–4 h (44). In the present report, although formalin fixation in paraffin-embedded samples posed challenges in accurately identifying bacterial organisms, the morphological characteristics observed through H&E staining suggested the presence of *Streptococcus* spp. or *Staphylococcus* spp. These bacteria, known as significant inducers of NETs, were observed in areas of bacterial vasculitis in this patient, aligning with findings from previous studies.

Extracellular histones, particularly H3 and H4 in NETs, are potential biomarkers and therapeutic targets for sepsis and inflammation. They induce cytotoxicity in endothelial cells and lethal effects in mice by promoting neutrophil margination and macro- and microvascular thrombosis. Thus, antibodies targeting histones could reduce mortality in sepsis models. Heparin, a widely available anticoagulant in veterinary medicine, has been shown to neutralize histone cytotoxicity, reducing inflammation and thrombosis. Its administration has demonstrated protective effects against histone-induced endothelial damage, making it a potential therapeutic option for sepsis-induced thrombosis (59). Additionally, activated protein C (APC), known for its antithrombotic, anti-inflammatory, and profibrinolytic properties, cleaves histones, reduces cytotoxicity, and improves microcirculation. While APC is not commonly used in veterinary medicine, its benefits in reducing mortality in sepsis models have been observed in various studies (60). Co-administration of APC with *E. coli* in baboons or histones in mice has shown mortality prevention (13). Therapeutic options targeting NETs, such as DNases with fewer side effects, are available in human medicine and warrant testing in veterinary medicine (12). Dornase alfa^29^, a recombinant human deoxyribonuclease I, acts as a mucolytic agent in severe COVID-19 by cleaving extracellular chromosomal DNA from NETs and cell-free DNA (61). Therefore, targeting the extracellular histones and DNA in NETs during leukocyte activation, sepsis, and other inflammatory conditions may prevent thrombosis, suggesting NET regulation as a novel treatment for bacterial vasculitis-induced thrombosis.

Bilateral partial amputation in veterinary medicine involves ethical considerations, particularly regarding outcomes and the potential role of prosthetics. Recent studies show that, although complications may occur, many animals adapt well post-surgery, with owners generally satisfied with the results, especially regarding mobility and quality of life (62). Advances in prosthetic technology now offer pets improved function, allowing them more active lifestyles, and reducing complications associated with limb loss. These developments support bilateral amputation as a viable alternative to euthanasia in select cases.

This study has limitations. First, this dog could have polyarteritis nodosa (PAN), a necrotizing arteritis that affects medium or small arteries and is characterized by a lack of association with ANCA. The presence of bacteria might have been secondary to extensive necrosis. PAN manifests with multisystemic symptoms, commonly affecting peripheral nerves and skin, with higher mortality linked to involvement of the gastrointestinal tract, kidneys, heart, and central nervous system, which mirrors the findings in this case. In veterinary medicine, the classification of vasculitis primarily focuses on histological patterns and lacks a comprehensive system that integrates etiology, pathology, and current biomarkers. Secondly, the patient exhibited dyspnea prior to euthanasia. Given that PAN is a systemic necrotizing vasculitis affecting multiple organs, a postmortem cardiovascular evaluation was warranted for diagnostic clarification; however, this was not possible due to client refusal. Additionally, future research should consider exploring the presence of NETs in non-infectious vasculitis to further understand their role in various vascular inflammatory conditions.

In conclusion, this is the first reported case of limb ischemic necrosis in a dog with bacterial vasculitis and thrombosis. This is the first veterinary case of NETs detection in a thrombus due to an infectious cause, indicating an immune response to infection. The potential involvement of NETs in thrombosis due to systemic inflammation, such as vasculitis, has been identified in previous studies. Understanding the mechanism of NETosis is essential for the prevention and treatment of thrombosis.

## Data Availability

The original contributions presented in the study are included in the article/Supplementary material, further inquiries can be directed to the corresponding author.

## Footnotes

^1^Procyte Dx, IDEXX Laboratories, Westbrook, Maine, USA.

^2^Catalyst One, IDEXX Laboratories, Westbrook, Maine, USA.

^3^Freestyle blood glucose meter, Abbott Laboratories, Abbott Park, Illinois, USA.

^4^VetChroma, Anivet, Seoul, South Korea.

^5^TEG 5000, Haemostasis Analyzer, Haemoscope Corporation, Niles, IL.

^6^SNAP 4Dx test, IDEXX Laboratories, Westbrook, Maine, USA.

^7^Antinuclear antibody (ANA) testing, IDEXX Laboratories, Westbrook, Maine, USA, was performed using indirect immunofluorescence assay (IFA).

^8^Antineutrophil cytoplasmic antibody (ANCA) testing, GC Labs (Green Cross Medical Foundation), Yongin, Korea, was conducted using fluorescent antibody test.

^9^Metronidazole (Metrynal Injection), Daehan Pharmaceutical Corporation, Seoul, South Korea.

^10^Prednisolone (Sorondo), Yuhanyanghaeng, Seoul, South Korea.

^11^Cyclosporine (Cyprolene), Jonggeundang, Seoul, South Korea.

^12^Pantoprazole (Pantolain), Dong-A ST, Seoul, South Korea.

^13^Pentoxifylline (Trental), Dong-A ST, Seoul, South Korea.

^14^Cefazolin (Cefazolin), Korean Koros Pharmaceutical, Seoul, South Korea.

^15^Famotidine (Gaster), Dong-A ST, Seoul, South Korea.

^16^Butorphanol (Butophan), Myungmoon Pharmaceutical, Seoul, South Korea.

^17^Midazolam (Midacom), Myungmoon Pharmaceutical, Seoul, South Korea.

^18^Propofol (Anepol), Hana Pharm, Seoul, South Korea.

^19^Fentanyl Injection, Hana Pharm, Seoul, South Korea.

^20^Lidocaine, Daehan Pharmaceutical Corporation, Seoul, South Korea.

^21^Cefotaxime, PharmGen Science, Seoul, South Korea.

^22^Niacinamide (Vitamin B3), Solgar, New Jersey, USA.

^23^Fish-derived omega-3 fatty acids (Duoomega), Kandiyoli, Istanbul, Turkey.

^24^Vitamin E (grandpherol soft cap), Yuhan Corporation, Seoul, South Korea.

^25^Doxycycline (Vydoxie), Medica Korea Co., Ltd., Seoul, South Korea.

^26^Rivaroxaban (Xarelto), Bayer, Leverkusen, Germany.

^27^Clopidogrel (Ropirel), CMG Pharmaceutical, Seoul, South Korea.

^28^Fluorescence microscope, Leica SP8 Confocal or Leica DMR Widefield, Leica Microsystems, Wetzlar, Germany.

^29^Dornase alfa (Pulmozyme soft cap), Genentech, South San Francisco, CA, United States.
