# Supplementary material for: Case report: In vivo detection of neutrophil extracellular traps in a dog with thrombosis induced by bacterial vasculitis
Source: Front Vet Sci. 2025 Feb 12;12:1470605. doi: 10.3389/fvets.2025.1470605 (PMC11862914; doi:10.3389/fvets.2025.1470605)
Supplement: Supplementary file 1 [file Table_1.DOCX]

**Immunofluorescence of Tissue Sections**

After dewaxing and rehydration, sections were incubated in HIER buffer (TRS, pH 9, Dako/10% glycerol) for 15 minutes in a steam cooker (Braun). After antigen retrieval, the sections were cooled in the HIER buffer at room temperature until the temperature dropped below 40°C. The sections were then rinsed three times with deionized water and once with TBS (pH 7.4), followed by permeabilization for 2 minutes with 0.1% Triton X-100 in TBS at room temperature. This was followed by three additional rinsing steps with TBS.

Sections were encircled with a PAP pen and treated with blocking buffer for 30 minutes to prevent nonspecific binding. The blocking buffer consisted of TBS supplemented with 1% BSA, 5% donkey normal serum, 5% cold water fish gelatin, 0.05% Tween 20, and 0.05% Triton X-100.

Primary antibodies were diluted in the blocking buffer as follows:

Mix 1: Rabbit anti-MPO (Dako AF3667), rabbit anti-neutrophil elastase (Abcam 68672, predicted to cross-react with dog), and rabbit anti-neutrophil elastase (Merck Millipore 481001), each diluted 1:100.

Mix 2: Sheep anti-neutrophil elastase (LS-B 4244), diluted 1:200, and rabbit anti-citrullinated H3 (Abcam 5103) and rabbit anti-citrullinated H4 (Millipore 07-596), each diluted 1:100.

Tissue sections were incubated with primary antibodies overnight at room temperature, which reflects an optimized protocol compared to the standard incubation at 4°C.

Secondary antibodies, raised in donkey and pre-absorbed against serum proteins from multiple host species (Jackson ImmunoResearch), were applied for signal detection. The secondary antibodies were diluted 1:500 in the blocking buffer.

Counterstaining with DAPI was performed using a 1 µg/mL DAPI solution included in the mounting medium (ProLong Gold Antifade Mountant with DAPI, Invitrogen P36931).
